# Virtual Staining, Segmentation, and Classification of Blood Smears for Label-Free Hematology Analysis

**DOI:** 10.34133/2022/9853606

**Published:** 2022-07-01

**Authors:** Nischita Kaza, Ashkan Ojaghi, Francisco E. Robles

**Affiliations:** ^1^School of Electrical and Computer Engineering, Georgia Institute of Technology, Atlanta, Georgia, USA; ^2^Wallace H. Coulter Department of Biomedical Engineering, Georgia Institute of Technology and Emory University, Atlanta, Georgia, USA

## Abstract

*Objective and Impact Statement*. We present a fully automated hematological analysis framework based on single-channel (single-wavelength), label-free deep-ultraviolet (UV) microscopy that serves as a fast, cost-effective alternative to conventional hematology analyzers. *Introduction*. Hematological analysis is essential for the diagnosis and monitoring of several diseases but requires complex systems operated by trained personnel, costly chemical reagents, and lengthy protocols. Label-free techniques eliminate the need for staining or additional preprocessing and can lead to faster analysis and a simpler workflow. In this work, we leverage the unique capabilities of deep-UV microscopy as a label-free, molecular imaging technique to develop a deep learning-based pipeline that enables virtual staining, segmentation, classification, and counting of white blood cells (WBCs) in single-channel images of peripheral blood smears. *Methods*. We train independent deep networks to virtually stain and segment grayscale images of smears. The segmented images are then used to train a classifier to yield a quantitative five-part WBC differential. *Results.* Our virtual staining scheme accurately recapitulates the appearance of cells under conventional Giemsa staining, the gold standard in hematology. The trained cellular and nuclear segmentation networks achieve high accuracy, and the classifier can achieve a quantitative five-part differential on unseen test data. *Conclusion*. This proposed automated hematology analysis framework could greatly simplify and improve current complete blood count and blood smear analysis and lead to the development of a simple, fast, and low-cost, point-of-care hematology analyzer.

## 1. Introduction

Hematological analysis assesses changes in the morphological, molecular, and cytogenetic properties of blood cells, in addition to blood cell enumeration. It is integral to diagnose and monitor a range of blood conditions and diseases, such as infections [1, 2], sepsis [3, 4], autoimmune diseases [5, 6], and different types of cancers [7, 8]. The typical workflow consists of collecting a peripheral blood specimen and analyzing it using a hematology analyzer to obtain a complete blood count (CBC), which includes red blood cell (RBC) and platelet counts, white blood cell (WBC) differentials (neutrophil, eosinophil, basophil, lymphocyte, and monocyte counts), and hemoglobin (Hb) levels [9]. Although modern analyzers are capable of automated analysis, they are expensive and bulky, use many chemical reagents, and require frequent calibration. Furthermore, cellular morphology often needs to be evaluated by a trained expert via manual microscopic examination. Microscopic examination of peripheral blood is performed by preparing a blood smear that is then fixed and stained, typically using Romanowsky-type stains (including Giemsa), which are generally composed of a basic dye that stains the nuclei and an acidic dye that acts as a counterstain [9]. Thus, hematological analysis is resource-intensive and time-consuming, requires trained personnel, and is susceptible to variability in staining.

Label-free techniques for quantitative analysis can address many of the limitations of conventional methods by eliminating the need for staining or exogenous contrast agents, thereby simplifying and speeding up the workflow. Several such techniques have been explored, including hyperspectral imaging [10], Raman microscopy [11], fluorescence lifetime imaging microscopy [12], and quantitative phase imaging [13–16]. While each method has its own unique advantages and disadvantages, there is a trade-off between the information provided by each method and its cost, complexity, and speed.

Deep-ultraviolet microscopy (UV) is a label-free imaging technique that leverages the distinctive spectral properties of endogenous biomolecules in this region of the spectrum (200-400 nm) to yield quantitative molecular and structural information from biological samples [17–22]. Owing to the shorter wavelength of UV light, deep-UV microscopy offers higher spatial resolution than conventional methods. Additionally, contiguous imaging of live cells is possible over long durations (>6 hrs) without significant photodamage [17]. These properties make deep-UV microscopy well suited to serve as a simple, fast, and low-cost alternative to modern hematology analyzers [21]. We recently developed a multispectral UV microscope [21] that enables high-resolution imaging of live, unstained whole blood smears at three discrete wavelengths: 260 nm (corresponding to the absorption peak of nucleic acids), 280 nm (corresponding to the absorption peak of proteins), and 300 nm (which does not correspond to an absorption peak of any endogenous molecule and can act as a virtual counterstain) [17–19, 21, 23]. We also introduced a pseudocolorization scheme that uses the multispectral UV images at these three wavelengths to generate images whose colors effectively recapitulate those produced by Giemsa staining and can thus be used for visual hematological analysis [21]. In addition, we demonstrated a five-part WBC differential by utilizing structural and molecular information at 260 nm in manually segmented cells [21]. We also introduced a color-based automated segmentation framework to segment WBCs from the pseudocolorized images [24].

In this work, we take advantage of the capabilities of deep learning for segmentation [25, 26], classification [15, 27, 28], and image-to-image translation [29–32] of label-free microscopy images, to develop an automated hematology analysis framework that operates on single-channel UV images acquired at 260 nm (having inherent nuclear contrast due to the absorption peak of nucleic acids), enabling simpler instrumentation and a factor of three improvement in imaging speed without sacrificing accuracy. Our virtual staining scheme accurately mimics the colors produced by the gold-standard Giemsa staining using only a single-channel image (single-wavelength imaging instead of multispectral imaging), unlike the pseudocolorization scheme introduced previously [21]. The problem of virtually staining a single-channel image is inherently ill-posed because it entails successfully inferring three different values (R, G, and B intensity values per pixel) solely from a single grayscale value. Based on the previous successes of generative adversarial networks (GANs) [33] (a special class of DNNs) in solving image-to-image translation problems [29, 34], we propose GAN-based virtual staining that enables fast, fixative-free, and label-free visual inspection of blood smears.

While several methods for segmentation and classification of WBCs have been proposed, most of them rely on feature extraction or training DNNs using stained images [35–39] or fail to provide an accurate five-part white blood cell differential [15, 16, 27, 28, 40]. Here, we present a segmentation method that uses only grayscale images (and is independent of the virtual staining branch of the pipeline) and has very high accuracy, with an average dice score of 0.9899 for cellular segmentation and 0.9718 for nuclear segmentation on an unseen test dataset. Segmentation is followed by a simple and fast classification technique that requires neither manual feature engineering nor long training times and achieves five-part WBC classification with an accuracy of 94.02% on unseen test data. Finally, we show that the combined segmentation and classification pipeline yields accurate differential cell counts.

## 2. Results

### 2.1. Deep-UV Microscopy of Live, Unlabeled Blood Cells from Whole Blood Samples

**Figure 1:**
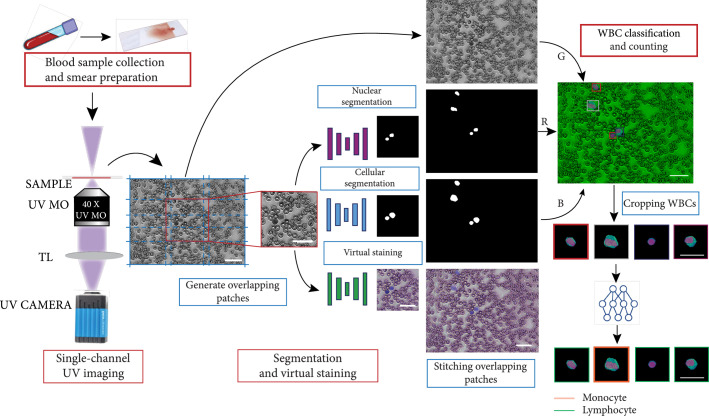
Workflow for deep-UV microscopy of blood smears, virtual staining, WBC segmentation, and classification. Whole blood smears are prepared on a quartz slide and imaged using the deep-UV microscopy system. The single-channel image is divided into overlapping patches that can be simultaneously virtually stained and segmented via three independent neural networks. The output patches are stitched together, and the segmentation outputs and the grayscale image are combined to generate a 3-channel image, from which WBCs are cropped and classified (scale bars: 30 *μ*m).

As shown in Figure 1, whole blood was collected from 23 healthy donors and patients according to protocols approved by the Institutional Review Board of Georgia Institute of Technology and Emory University. Blood smears are prepared on quartz microscope slides using 10 *μ*L of whole blood, without any cell fixation, dilution, or staining. As soon as a smear dries, it is imaged with the UV microscopy system. The sample is raster scanned to acquire a grid of overlapping UV image tiles (each having a field of view (FOV) of 170 μm×230 μm) spanning a 1 mm×2 mm FOV in total, to have sufficient cells (>20,000) for statistically significant cell counts and reliable diagnosis. The image tiles from all 23 individuals were divided into smaller image patches to train and validate the deep networks for automated analysis. Since basophils and eosinophils only occur in small numbers in the smear images, images of granulocytes (i.e., neutrophils, eosinophils, and basophils) that were isolated using a magnetic antibody-based selection technique were included in the training and testing of our networks (more details in Materials and Methods).

Our analysis framework operates on the single-channel, grayscale image tiles that are divided into smaller overlapping patches for network prediction, following which the output patches are stitched back together (see Figure 1). The segmentation masks are combined with input image tile, to generate an RGB image tile from which patches containing WBCs can be extracted for classification. For visual hematology analysis and cell counting, the RGB image tiles containing the segmentation masks and the virtually stained image tiles can be stitched into large images spanning the full 1×2 mm FOV.

### 2.2. Virtual Staining of Single-Channel Deep-UV Microscopy Images

**Figure 2:**
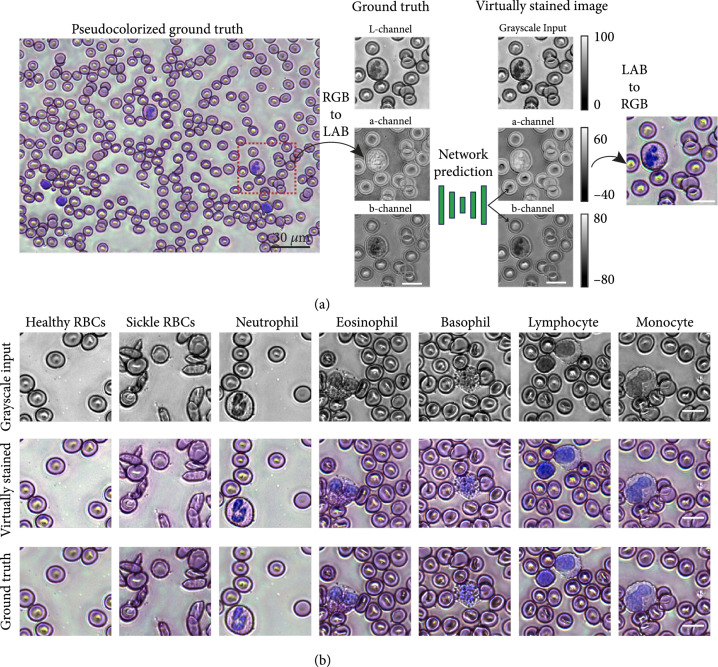
(a) Virtual staining in the LAB color space. A patch extracted from the pseudocolorized image serves as the ground truth. The network predicts only the “a” and “b” channels, and the input grayscale image is treated as the output L-channel. (b) Input grayscale (top row), virtually stained (middle row) and ground truth pseudocolorized image patches (bottom row) containing different types of cells (scale bars: 10 *μ*m.)

Our virtual staining scheme translates single-channel smear images (i.e., images acquired with single wavelength illumination using 260 nm light) of unstained live blood cells into colorized images, whose colors accurately recapitulate those of Giemsa stains. Virtual staining is performed through a conditional generative adversarial network (cGAN) trained using pairs of cropped image patches consisting of single-channel UV images and their corresponding pseudocolorized images, generated from multispectral UV microscopy data [21]. Training is performed in the Lab color space, an alternative to the RGB color space (where all three channels contain color information). In the Lab color space, the intensity (the grayscale image) is encoded by the luminance channel (L) and color information is encoded in the two other channels (“a” and “b”) as shown in Figure 2(a) (see Materials and Methods for more details). The Lab color space is chosen because a given change in the numerical values of the “a” and “b” channels corresponds to a similar perceived change in the color, resulting in smoother color transitions and fewer instances of abrupt changes in color due to a small change in the pixel values (unlike in RGB color space) [34]. Furthermore, this choice of color space leads to a simpler network with fewer parameters that better preserves structure in the final image since the input image is treated as the output L-channel. As shown in Figure 2(a), however, the grayscale input image is not identical to the *ground truth* L-channel and appears to have a slightly higher contrast. But rather than having a detrimental effect on the virtual staining, using the input image as the output L-channel causes the nuclear contrast to be enhanced in the final colorized output image. As in Figure 2(a), the virtually stained image has greater nuclear contrast and the nucleus appear to have a deeper blue hue. Figure 2(b) compares the grayscale inputs, the virtually stained images, and the ground truth pseudocolorized images for test image patches (from all 23 blood smears that are previously unseen by the network) containing healthy and sickle RBCs, and different types of WBCs. The grayscale images acquired at 260 nm have sufficient resolution and contrast to clearly capture the nuclear morphology and cytoplasmic features of different cells, which are essential for further analysis and quantification. The virtually stained images are in excellent agreement with the ground truth pseudocolorized images, and the performance of the virtual staining is quantified using the multiscale structural similarity index (MS-SSIM) [41], a modification to the single-scale structural similarity index (SSIM) that is more representative of perceived image quality. The MS-SSIM values averaged across ~3600 test image patches are 0.9408, 0.9155, and 0.8811 corresponding to the R, G, and B channels, respectively, with the average across the three channels being 0.9125. The more familiar SSIM [42] averaged across the same test dataset is 0.7811, with the SSIM of the R, G, and B color channels being 0.8687, 0.7751, and 0.6995, respectively. The lower MS-SSIM and SSIM values for the blue channel likely result from differences in the contrast of the grayscale input image compared to the *ground truth* L-channel, as explained previously. We also note that small changes in the color values of the background region (that do not affect the appearance of cells) can have a pronounced effect on the SSIM, whereas the MS-SSIM is less affected by such imperceptible variations.

**Figure 3:**
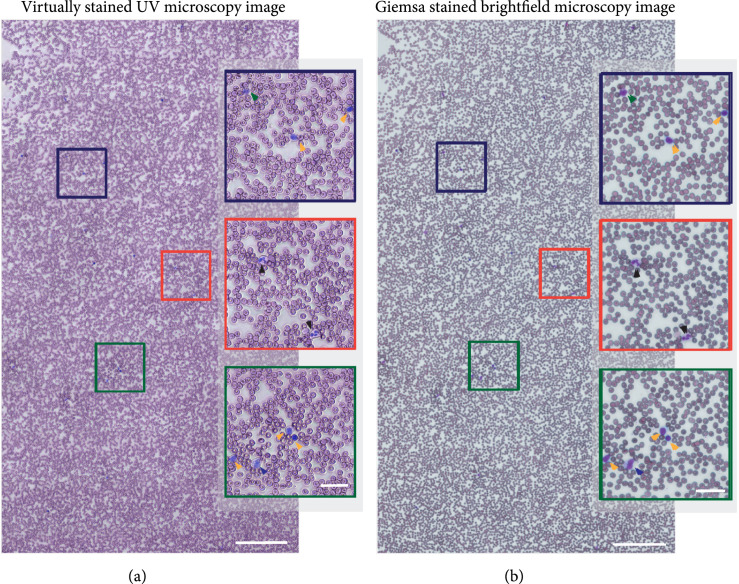
(a) Wide-field virtually stained UV image of a whole blood smear from a healthy donor. (b) Corresponding white-light bright-field microscopy image after Giemsa staining (scale bars: 200 *μ*m). The magnified insets highlight cellular features, with black arrowheads pointing to neutrophils, yellow arrowheads showing lymphocytes, a blue arrowhead pointing to a monocyte, and a green arrowhead pointing to a basophil (inset scale bars: 30 *μ*m.)

Figure 3 compares a large virtually stained image (FOV ~1 mm×2 mm) obtained by stitching virtually stained image tiles (~170 μm×230 μm) (Figure 3(a)) to a Giemsa-stained smear imaged with bright-field microscopy (Figure 3(b)). Such large area blood smear images can be useful for visual hematology analysis. As the figure shows, our virtually stained images reproduce the critical features of different blood cells that are seen in the stained smear image with high fidelity. The virtually stained images clearly highlight the distinct nuclear morphology of different WBCs and accurately portray the appearance of red blood cells (normal and sickled). The granularity of all granulocytes is preserved, partially due to the strong scattering of the granules at UV wavelengths. Our current training dataset contains a small number of eosinophils and basophils leading to a subtle difference in the hue of the eosinophils compared to ground truth Giemsa-stained images. Specifically, our single-channel virtual staining has a less vibrant orange-ish hue in the cytoplasm of eosinophils compared to ground truth. Nevertheless, this does not affect further quantitative analysis since both the segmentation and classification do not depend on the virtually stained images. Moreover, the virtually stained images can be enhanced based on the segmentation and classification to better resemble the ground truth for visual analysis (see Figure S1). We further note the presence of subtle halo-like structures in the UV images due to diffraction and scattering at cell edges and the fact that our light source has a fairly high degree of spatial coherence. However, this does not strongly affect the cells’ appearance or the qualitative and quantitative analyses that follow.

### 2.3. Cellular and Nuclear Segmentation

**Figure 4:**
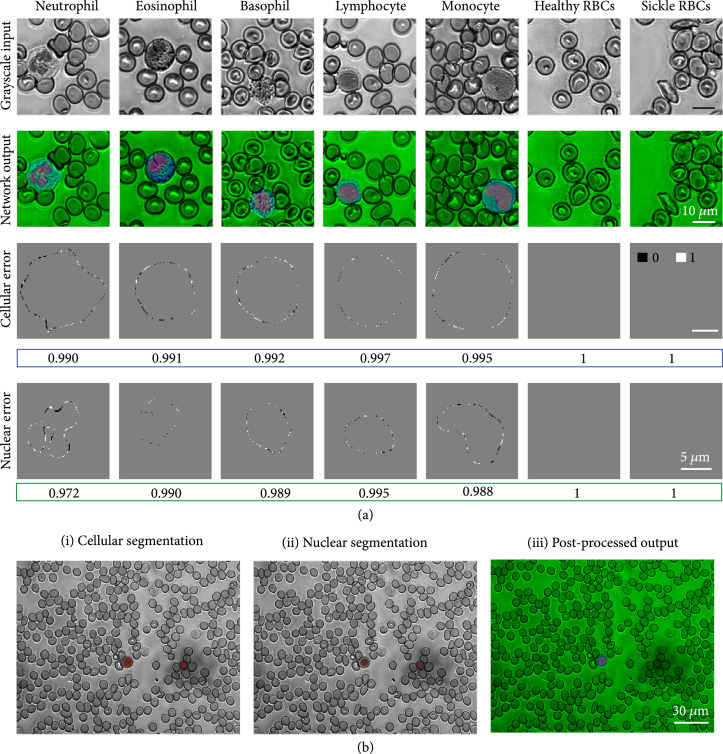
(a) Input grayscale image patches (top row) and corresponding combined cellular and nuclear segmentation results (second row) containing different types of cells. Error images for cellular (third row) and nuclear (fourth row) segmentation show the error with respect to the ground truth masks used for training. The Dice coefficient is listed below each image. (b) Raw cellular and nuclear segmentation (i and ii) masks contain errors which are removed by postprocessing (iii).

Our framework to automatically segment WBCs from single-channel UV microscopy images makes use of two independent convolutional neural networks (CNNs) having identical architectures, to predict cell masks and nuclear masks from input grayscale images. Figure 4(a) shows network predictions on test image patches (that are previously unseen by the network). The cellular and nuclear segmentation masks are overlaid on the grayscale image as shown in Figure 4 and demonstrate the extremely accurate and robust segmentation capabilities for different types of WBCs. The error images, obtained by subtracting the network outputs from the ground truth, show near-perfect segmentation masks, as is also evident from the Sørensen–Dice coefficient values being very close to one for both the cellular and nuclear masks. The Sørensen–Dice coefficient or the Dice coefficient is a measure of similarity between two samples, and the metric takes a maximum value of one. The Dice coefficient averaged across the entire test dataset of 2010 images (across all smear samples) is 0.9899 for cellular segmentation and 0.9718 for nuclear segmentation, further validating the accuracy of our segmentation method.

For segmentation of UV image tiles, each tile is divided into nine overlapping image patches, and the corresponding masks are stitched back together following network prediction. The cellular and nuclear masks are jointly postprocessed (i.e., cell regions without a corresponding nucleus and nuclear regions without a corresponding cell are removed) to minimize segmentation errors. Segmentation errors are generally a result of inconsistencies in the background, as shown in Figure 4(b). The postprocessing scheme effectively deals with erroneous pixels and is based on simple morphological operations and thresholds based on proprieties such as the mean intensity and the areas of the cell and nucleus (see Materials and Methods for details).

### 2.4. WBC Classification and Counting

**Figure 5:**
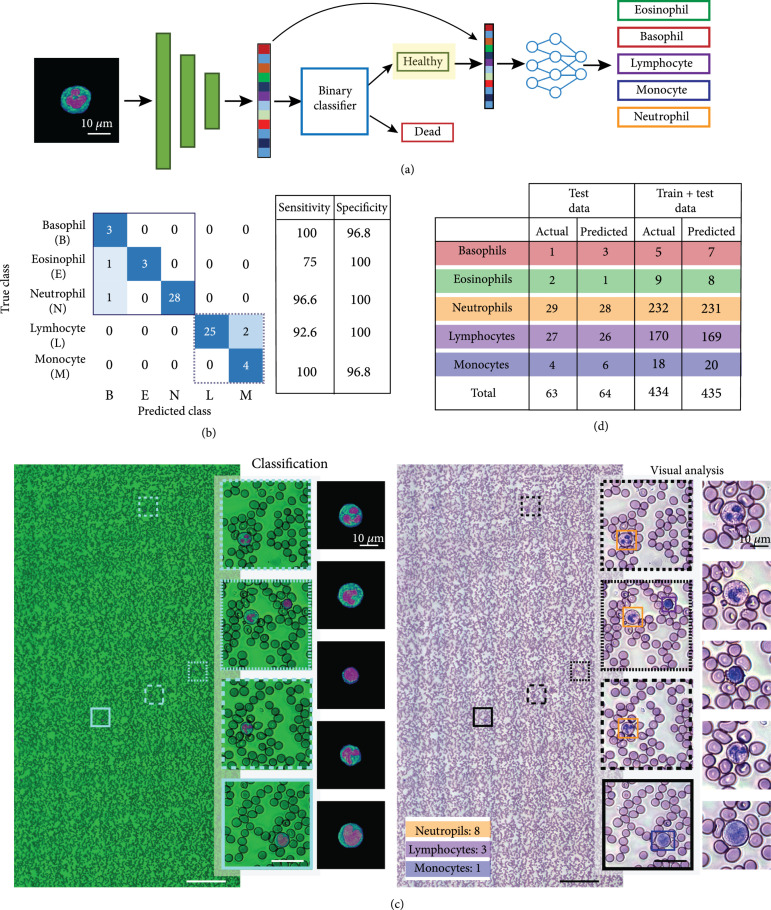
(a) Two-step classification procedure. Three-channel images generated after segmentation are passed to a pretrained network for feature extraction. The extracted features are used to classify the cell as healthy or dead, and the healthy cells are classified into one of the 5 WBC subtypes. (b) Confusion matrix, sensitivity and specificity for five-part classification on test data from 5 blood smear samples. (c) Cell classification and counting in wide-field images (scale bars: 200 *μ*m). Patches containing WBCs are extracted from the wide-field image, classified, and counted. Classification results are juxtaposed with the virtually stained image for visual analysis (inset scale bars: 15 μm.). (d) Ground truth (actual) and predicted cell counts from the 5 test blood smear samples (test data), as well as all 23 samples (test+train data).

Differential cell counts are an indispensable quantitative metric for disease diagnosis and monitoring. While hand-crafted features can be very effective in classifying small datasets, they may not generalize as well to larger, more diverse datasets [40]. Deep neural networks can potentially achieve better performance, but require a large labeled dataset for training [40, 43]. Here, we take advantage of transfer learning [44] to achieve robust classification on a small dataset (~500 cells in total). A pretrained ResNet-18 [45] trained on the ImageNet dataset [46] was used as a fixed feature extractor to extract features from a three-channel image (consisting of the segmentation masks and the grayscale input) as shown in Figure 5(a). ResNet-18 is chosen for its extremely efficient feature representation and faster inference speeds compared to several other state-of-the-art networks [47]. In keeping with current clinical practice, dead WBCs must be omitted from cell counts, so we use a two-stage classification procedure that first eliminates dead cells and then classifies WBCs into the five subtypes (neutrophils, basophils, eosinophils, lymphocytes, and monocytes) (Figure 5(a)).

We first trained a binary classifier on segmented cells from blood smear images of 18 individuals to distinguish between healthy and dead cells. The trained binary classifier was tested on smear data from 5 different (unseen) individuals, to ensure that our classifier is robust to cross-donor variability in WBC morphology. An additional checkpoint was added to correctly classify healthy cells that were misclassified as dead cells (see Materials and Methods). The overall classifier had an accuracy of 98.76%, misclassifying a single dead cell as a healthy cell (out of 81 test cells). We then trained a five-part classifier on segmented WBCs from blood smear images of 18 individuals and tested on segmented WBCs from blood smears images from 5 different individuals. Since both the test and train datasets contained small numbers of basophils and eosinophils, images of magnetically isolated granulocytes were added to both datasets. We achieved a test accuracy of 94.02%, with 4 of the 67 cells misclassified, and a high sensitivity (above 90% for all classes except eosinophils) and specificity >96% for all classes (Figure 5(b)). Again, the low sensitivity to eosinophils is a result of the low number of cells in the training and test data set and can be improved with additional data. From the confusion matrix in Figure 5(b), we see that the misclassified eosinophil and neutrophil are classified as basophils, likely because of the similarity in nuclear morphology and the presence of cytoplasmic granules in all granulocytes. Along similar lines, larger lymphocytes may be misclassified as monocytes due to similarities in size and cytoplasmic features.

The trained classifiers were then used to obtain differential cell counts from all the samples. The RGB image tiles (consisting of the grayscale image and the postprocessed segmentation masks) are stitched to generate a single wide-field image, from which patches containing WBCs are extracted (so as to avoid counting cells in overlapping regions of tiles multiple times) as shown in Figure 5(c). The cells are classified and counted, and the labels (indicated by colored bounding boxes corresponding to different cell types) are overlaid on the virtually stained image for visual analysis. The total cell counts obtained from the 5 test samples as well as from all 23 samples are given in Figure 5(d). The ground truth (actual) counts are from visual assessment by trained personnel while the predicted counts are a result of automated segmentation, classification, and counting. The predicted and actual counts are in excellent agreement, indicating the reliability of the combined segmentation and classification pipeline.

## 3. Discussion

We have introduced an automated pipeline for hematological analysis based on single-wavelength (260 nm) deep-UV microscopy. Our approach is label-free and fixative-free, relying only on the UV absorption of endogenous biomolecules (e.g., nucleic acid, proteins, and hemoglobin), thereby rendering the chemical reagents used in traditional hematology analysis workflows redundant. We note that the brief UV exposure (30–100 ms) in our approach does not cause photodamage or alter intracellular morphology [21], enabling subsequent analysis or validation by Giemsa staining or fluorescence-based techniques.

Our analysis pipeline requires only single-channel grayscale images (acquired at 260 nm), thus reducing the imaging time by a factor of three compared to using multispectral UV images. Additionally, the system can be further simplified, since the broadband source and filters can be replaced by a single 260 nm LED, and other low-cost alternatives can result in a very compact system (potentially portable) that costs <$5000, compared to commercial hematology analyzers that are extremely large and cost between $80,000 and $120,000. Commercial analyzers also have higher operating costs, requiring many reagents (>10) and regular calibration and maintenance, whereas our deep-UV microscopy-based assay requires no additional sample preparation, reagents, or calibration. Further, compared to the RGB cameras used for bright-field microscopy of stained smears, single-wavelength imaging with a monochrome camera takes advantage of the higher space-bandwidth product of the camera and yields better resolution.

The entire pipeline is fast and efficient. Imaging protocols take ~3 minutes for a grid of image tiles spanning a 1 mm×2 mm area (containing >20,000 cells) and can be concurrent with image processing. Segmentation and classification of all the image tiles take ~2 minutes (on a GPU-enabled computer with an Intel Core i7-7800X CPU and a NVIDIA GeForce GTX 1080 Ti GPU), which is well suited for point-of-care, low-resource settings, and potentially at-home use. The virtual staining scheme is also relatively fast, currently taking a little over 4 minutes to virtually stain all the image tiles in a sample. Stitching of the image tiles for virtual hematology analysis takes a little over 2 minutes, resulting in a total time of 6-7 minutes for virtual staining, compared to conventional Giemsa staining protocols that usually take over 30 minutes. Note, however, that virtual staining and stitching are only necessary for visual assessment by experts and can be omitted if cell counts are the only parameter of interest (as is the case for most applications). Thus, our fast and easy segmentation, classification, and virtual staining scheme is well suited for translation into clinical, point-of-care, at-home, and low-resource settings.

As we show here, our virtual staining scheme transforms grayscale images into colorized images whose colors recapitulate those observed with the gold-standard Giemsa stains with high fidelity. While several virtual staining techniques based on a variety of label-free imaging techniques have been presented [29–32], they are mostly geared toward the staining of tissues for histopathology and are not designed to digitally stain and analyze blood smears. Further, our segmentation method is robust and achieves comparable or even better performance than methods based on stained or pseudocolorized images, without the need for fixing and staining the sample [36–38] or the need for multispectral imaging [24]. We have presented a simple and robust classification and counting procedure that utilizes cellular and nuclear segmentation masks along with the grayscale images to first exclude dead WBCs and then classify healthy WBCs into five subtypes. Thus, we achieve a five-part WBC differential, which is integral to diagnose and monitor many blood diseases and conditions. The proposed deep learning-based classifier enables more accurate neutrophil classification (with an accuracy of 96.5%) compared to our previous approach based on feature engineering (with an average test accuracy of $\left.91.9\right)\%$ [24]. Our classification accuracy is limited by the size of our dataset, particularly the small numbers of monocytes, eosinophils, and basophils, but can be readily improved with more data. Despite the limited dataset, the performance of our combined segmentation and classification scheme based on fixative-free and label-free images is comparable to other methods based on stained images [37, 40]. Similarly, other label-free techniques achieve comparable performance for the classification of certain WBC subtypes, but require additional sample preparation or isolation [15, 16] or bulky and expensive instrumentation [27, 28] compared to our proposed framework. As mentioned above, our automated analysis pipeline is fast, taking approximately 2 minutes for the complete analysis of one sample (>20,000 cells) including segmentation, classification, and counting, allowing nearly real-time analysis. Segmentation and postprocessing of the image tiles take nearly 1.5 minutes. The duration of the classification step depends on the number of cells in our sample, but is very fast and takes less than 20 s for any of the samples in our dataset. The speed of the analysis can be further improved by processing multiple image tiles in parallel. Since the virtual staining is completely independent from the segmentation and classification, it can be performed in parallel or can be omitted entirely if visual inspection of the blood smear is not necessary. Finally, given the fast pace of development of deep neural networks, it is possible that future architectures may continue to improve colorization, segmentation, and classification accuracy. Nevertheless, here we have shown that our simple, low-cost, and fast UV method, coupled with efficient deep networks, can achieve a five-part WBC differential, which is integral to diagnose and monitor many blood diseases and conditions.

In conclusion, we leverage the high-resolution, quantitative, label-free, molecular imaging capabilities of deep-UV microscopy to enable low-cost, fast, and automated hematology analysis. Our pipeline yields virtually stained images for visual hematology analysis as well as differential cell counts in a matter of minutes from single-channel grayscale images. Thus, our analysis pipeline offers substantial improvements over conventional hematology analysis workflows and can be very beneficial for point-of-care, at-home, or low-resource settings. Our automated analysis can be coupled with a microfluidic device to develop a single-wavelength-based compact, fully automated, label-free, point-of-care-hematology analyzer and will form part of our future work.

## 4. Materials and Methods

### 4.1. Preparation of Whole Blood Smears

Whole blood collected from healthy donors or patients was added to an anticoagulant solution (sodium citrate; Becton Dickinson), and blood smears were prepared on uncoated quartz slides using 10 μL of whole blood. Blood samples were collected from 23 individuals (4 healthy donors, 4 patients with sickle cell disease, 4 patients with thrombocytopenia, and 11 patients with neutropenia). All protocols were approved by the Institutional Review Boards of Georgia Institute of Technology and Emory University, and informed consent was obtained from the donors. After drying the samples in air for 5 minutes, UV imaging was performed.

### 4.2. Experimental Setup

The deep-UV microscopy system was illuminated by a broadband laser-driven plasma light source (EQ-99X LDLS, Energetiq Technology), whose output light was collected through an off-axis parabolic mirror (Newport Corporation) and relayed to the sample using a short-pass dichroic mirror (Thorlabs, NJ, USA). UV band-pass filters centered at 260, 280, and 300 nm (Chroma Technology Corp, VT, USA) were installed on a filter wheel for multispectral imaging (the light intensity on the sample plane was measured to be 0.14, 4.5, and 0.22 mW at 260, 280, and 300 nm, respectively); only the 260 nm filter is necessary for single-channel imaging. A 40x UV microscope objective (NA 0.5) (LMU-40X, Thorlabs), which achieves an average spatial resolution of ~280 nm, was used for imaging. Images were captured using a UV-sensitive CCD (pco.ultraviolet, PCO AG, Kelheim, Germany) camera (integration time is typically between 30 and 100 ms). with each pixel covering an approximate area of 0.165 μm×0.165 μm on the sample. A high-precision, three-axis motorized stage (MLS2031, Thorlabs) was used to focus and raster scan the sample to acquire a series of UV image tiles (each having a FOV of ~170 μm×230 μm) that span a 1 mm×2 mm FOV in total. The total imaging time was approximately three minutes (per wavelength) and is limited by the translation stage.

### 4.3. Preliminary Data Processing

Each image tile in the series, obtained by raster scanning the sample at a particular wavelength, was normalized by a background image acquired at the same wavelength, to minimize any illumination artifacts. The background image was acquired at a blank region of the sample, keeping all other conditions unchanged. While the automated pipeline presented in this work relies only on a single-channel UV microscopy image acquired at 260 nm, multispectral UV imaging (at 260, 280, and 300 nm) was required to generate the pseudocolorized images [21] that serve as the ground truth to train our virtual staining network. The corresponding images at the three wavelengths were registered using an intensity-based registration algorithm and pseudocolorized as described in Ref. [21].

### 4.4. Deep Learning-Based Virtual Staining

Single-channel UV microscopy images (input) and their corresponding pseudocolorized images (ground truth) were paired to train a cGAN for virtual staining. A GAN consists of two networks—a generator that generates new examples of data and a discriminator that attempts to distinguish the generated examples from the ground truth—that are simultaneously trained. Our network was trained in the Lab color space, where the color information is encoded in two channels (“a” and “b”) instead of three. The network is trained to predict the two color channels, which are then concatenated with the grayscale image (L- channel) to generate the colorized Lab image. Instead of using random noise as the input to the generator as in the case of a traditional GAN, we used a cGAN where the grayscale input serves as a prior for the “a” and “b” channel images predicted by the generator.

#### 4.4.1. Data Preparation

Twenty-four 256×256 pixel image patches were extracted with minimal overlap from each single-channel image tile (1040×1392 pixels, having a FOV of ~170 μm×230 μm), across all 23 blood smears. Patches containing no cells (only background) and patches with erroneous colorization were excluded. For our virtual staining scheme to be valid, it is imperative for WBCs to be correctly colorized and clearly distinguishable. Since the proportion of WBCs in the images is relatively small, the dataset was augmented with WBC images. This was done by using ground truth segmentation masks to detect WBCs in the image tiles and extracting overlapping patches containing WBCs. The same procedure was used to extract ground truth image patches from the pseudocolorized images, ultimately resulting in a dataset of ~74,000 image pairs (~3600 images were separated to serve as test data). The ground truth RGB images were converted to the Lab color space prior to training. Since there was some variations in the gray values across images from different samples, a simple preprocessing operation was applied to the input grayscale images. The preprocessing was in the form of a histogram operation applied to each image (normalized to 0-1 by dividing by its maximum pixel value) that saturates the top 1% and bottom 1% of the pixels and enhances contrast.

#### 4.4.2. Network Architecture and Training

**Figure 6:**
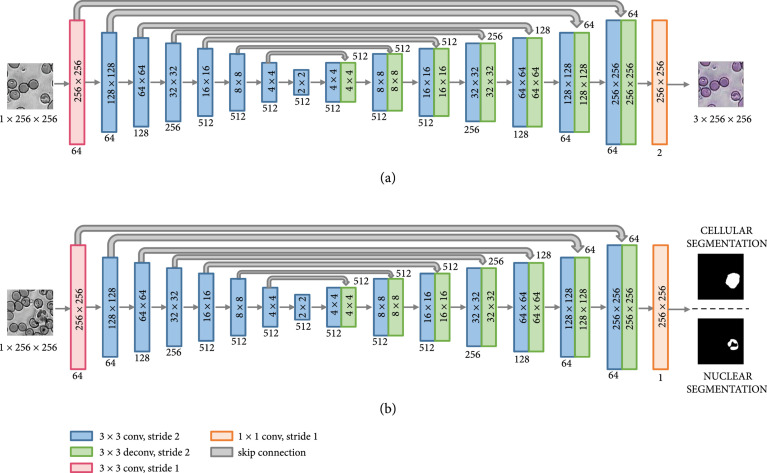
(a) Architecture of the generator used for virtual staining. (b) Architecture of the CNN used for cellular and nuclear segmentation.

The generator is a fully convolutional network with encoding and decoding paths with skip connections, that is based on the U-Net [48] (shown in Figure 6(a)) and is similar to the generator in Ref. [34]. In the encoding or downsampling path, 3×3 convolutional kernels were used with strided convolutions (stride of 2), followed by batch normalization and a leaky ReLU (LReLU) activation function with a slope of 0.2. The decoding path used 3×3 transposed convolutions with a stride of 2 to perform the upsampling, followed by batch normalization and a ReLU activation function. The architecture of the discriminator is similar to the encoding path of the generator and contains 4 convolutional layers (3×3 convolutional kernels were used with strided convolutions (stride of 2) as before) having 64, 128, 256, and 512 channels, respectively, followed by a fully connected layer with a sigmoid activation function [34].

We used a modified version of the GAN cost function [49] for a conditional GAN along with a total variation regularization term added to the generator loss function to ensure structural similarity [34]. A weight initialization similar to Ref. [50] and the Adam optimizers [51] (with $\beta_{1}=0.5$, $\beta_{2}=0.999$, and a learning rate of 0.0003) for both the generator and discriminator were used for training. We trained our model for 20 epochs with a batch size of 8, for approximately 60 hours and computed the L1 loss on the validation set after each epoch (see Figure S2). Our network was implemented in PyTorch 1.9.1 using a GPU-enabled computer (Intel Core i7-7800X CPU and NVIDIA GeForce GTX 1080 Ti).

#### 4.4.3. Postprocessing of Virtually Stained Images

Image patches were virtually stained by the trained generator in the Lab color space. The network outputs were then converted back to the RGB color space, followed by simple postprocessing operations to white balance the images. A constant value of 0.02 was added to each of the RGB channels to increase the brightness of the image patch. Each image patch was then converted to the HSV color space, where a constant (0.07) was added to the value (V) channel and then converted back to the RGB color space. The MS-SSIM and SSIM were calculated for all three color channels of each image patch, and the values were averaged to obtain a single MS-SSIM or SSIM value for every patch. Five scales were used to compute the MS-SSIM, and the SSIM values from each scale were averaged using weights from a Gaussian distribution.

#### 4.4.4. Virtual Staining for Visual Hematological Analysis

Each image tile acquired by the camera (1040×1392 pixels) was normalized by a blank background and contrast adjusted using a histogram operation as before. Owing to computational constraints, each image tile was divided into nine 512×512 pixel patches (the larger patch size offered a good trade-off between speed and accuracy) that were input into the trained generator. The virtually stained image patches were then stitched together by averaging overlapping regions, and postprocessed as explained previously. For visual hematology analysis, the image tiles (arranged in a grid of 13×9 images) were stitched into a single wide-field image using the Grid/Collection stitching plugin [52] of the Fiji [53] software. The plugin calculates the overlap between each tile and linearly blends the overlapping portions resulting in a single large image. An additional postprocessing step was added to the wide-field images to improve the contrast for visual analysis. MATLAB’s imadjust function was used to saturate the top and bottom 1% of the pixels in each of the color channels, with the minimum set to zero for all three channels, and the maximum set to 0.95 for the R channel, and 0.93 for the G and B channels.

### 4.5. Deep Learning-Based Cellular and Nuclear Segmentation

Two independent convolutional neural networks (CNNs) were trained to segment out cells and nuclei from single-channel UV microscopy images.

#### 4.5.1. Data Preparation

First, for ground truth segmentation, we leveraged our color-based segmentation scheme previously introduced in Ref. [24] to generate cellular and nuclear masks from the 3-wavelength, pseudocolorized image tiles (1040×1392 pixels) (whose 260 nm wavelength channel corresponds to the single-channel (grayscale) image tiles in this work). Some masks with erroneous pixels at the edges were manually reannotated using MATLAB’s *image segmenter app* to improve the quality of the ground truth available to the networks. Next, overlapping patches of 256×256 pixels containing WBCs, and a small number of patches containing no WBCs, were extracted from the larger grayscale tiles and masks. Additionally, since basophils and eosinophils only occur in small numbers in the smear images, images of granulocytes (20 images of each granulocyte subtype) that were isolated using a magnetic antibody-based selection technique [21] were added to the dataset. These grayscale images were manually annotated using MATLAB’s *image segmenter app* to generate ground truth cellular and nuclear masks as before. Since the isolated granulocytes were imaged with a slightly different magnification (having an effective pixel size of 0.127 μm×0.127 μm), the images and their corresponding masks were reinterpolated to be consistent with the smear images. The reinterpolated grayscale images and masks were flipped and rotated to further augment the dataset, yielding ~51,000 training images, ~13,000 validation images, and ~2000 test images. The grayscale images were preprocessed using the same histogram operation as before.

#### 4.5.2. Network Architecture and Training

The architecture of the CNNs used for segmentation is inspired by the U-Net [48], which is well suited for biomedical image segmentation, and is almost identical to that of the generator used for virtual staining (as shown in Figure 6(b)). The main differences are that the segmentation networks used ReLU activation functions in both the encoding and decoding paths and that they have single-channel outputs (instead of the two-channel output of the generator). The performance of our network is similar to the classical U-Net (a detailed comparison is presented in the Supplementary Materials), but is less computationally demanding, with fewer parameters (~23.6 million compared to ~31 million). The networks were trained using a combination of the binary cross-entropy and the dice loss [54]. The Adam optimizer [51] (with a learning rate of 0.005 that decayed by a factor of 0.2 when the loss plateaued) was used for training. Our cell segmentation network was trained for 80 epochs, and the model with the lowest loss on the validation dataset was chosen (see Figures S3(a) and S3(b)). The nuclear segmentation network was initialized with the trained weights of the cell segmentation network and trained for 95 epochs, with the best model chosen on the basis of the validation loss (see Figures S3(c) and S3(d)). Both models were implemented in PyTorch 1.9.1 and trained using a GPU-enabled computer (Intel Core i7-7800X CPU and NVIDIA GeForce GTX 1080 Ti) with a batch size of 8, for approximately 40 hours each.

#### 4.5.3. Prediction on Image Patches and Tiles

Test image patches (256×256 pixels) were segmented by the trained CNNs, and the Dice coefficient for cellular and nuclear segmentation was calculated for each image patch. The Dice coefficient was averaged across the entire test dataset of 2010 images. The larger image tiles were normalized, and contrast-adjusted image tiles were divided into nine 512×512 pixel patches (as in the case of virtual staining) that were input into the cellular and nuclear segmentation networks. The predicted binary mask patches were then stitched together, using a logical OR operation in overlapping regions.

#### 4.5.4. Postprocessing of Segmented Masks

The cellular and nuclear segmentation masks were jointly postprocessed to remove incorrectly segmented cells and some dead WBCs. A morphological opening operation was first performed on both masks to eliminate any groups of pixels with an area smaller than a typical cell. Cell regions without a corresponding nucleus and nuclear regions without a corresponding cell were removed. Some RBCs which were incorrectly segmented due to anomalous dark regions, and some dead WBCs in the background were excluded using thresholds based on the mean intensity, area, solidity, and Euler number (a topological property) of the cellular and nuclear masks. The postprocessing was performed in MATLAB.

### 4.6. Deep Learning-Based WBC Classification

Accurate classification of WBCs is necessary in order to obtain reliable differential cell counts. Here, we used a pretrained ResNet-18 as fixed feature extractor and then trained fully connected networks for five-class classification.

#### 4.6.1. Data Preparation

The postprocessed cellular and nuclear segmentation masks were combined with the grayscale image to generate a 3-channel RGB image (e.g., second row of Figure 4(a)) since Resnet-18 requires RGB inputs; the nuclear mask (multiplied by a factor of 0.5) was assigned to the red channel, the grayscale image, masked by the cell mask, was assigned to the green channel, and the cell mask (multiplied by a factor of 0.5) was assigned to the blue channel. WBCs were cropped (into 224×224 pixel patches) from all the image tiles across all the samples to generate a classification dataset. We note that overlap between image tiles resulted in more than one image of some cells (the repeated cells were retained in the training set but removed from the test set). The corresponding cells in the pseudocolorized images (that recapitulate Giemsa-stained images) were classified by a board-certified hematologist to provide ground truth labels. The dataset contained healthy WBCs, as well as some dead cells (postprocessing of our segmentation masks eliminated some but not all of the dead WBCs) that would need to be omitted from differential cell counts. The dead cells (~80 in total) were separated from the healthy cells and augmented by saving flipped and rotated versions of the images, in order to train a binary classifier. A training dataset was created with WBCs from 18 individuals whereas the test dataset contained cells from 5 different individuals. Due to the limited data available, a separate validation set was not used.

To deal with the small numbers of basophils and eosinophils in the smear images for the five-class classification, images of granulocytes (20 images of each granulocyte subtype) that were isolated using a magnetic antibody-based selection technique [21] were added to the dataset. As with the segmentation dataset, the images were reinterpolated to have the same magnification as the smear images. From our dataset of approximately 500 cells, WBCs from 5 individuals were separated for testing, as before. Since the test dataset contained only one basophil and two eosinophils, two images each of isolated basophils and eosinophils were added to the test dataset. Our train dataset was extremely unbalanced, with the number of lymphocytes and neutrophils far exceeding the numbers of eosinophils, basophils, and monocytes. Thus, the images in these three classes were augmented by saving flipped and rotated versions of each image, resulting in a more balanced training set.

#### 4.6.2. Deep Learning-Based Classifiers

As our classification dataset was relatively small, we opted for transfer learning from a pretrained network rather than training from scratch. We used a pretrained ResNet-18 [45] (ResNet-18 was chosen for its fast inference times and efficient feature representation) trained on the ImageNet dataset [46] as a fixed feature extractor as shown in Figure 5(a) to extract a feature vector containing 512 features from each image. Fully connected networks were implemented in PyTorch 1.9.1 and trained on these features for cell classification using a GPU-enabled computer (Intel Core i7-7800X CPU and NVIDIA GeForce GTX 1080 Ti).

In our two-step classification process, we first trained a binary classifier to classify cells as “healthy” or “dead.” The network consisted of an input layer with 512 inputs, four hidden layers with 128, 64, 32, and 8 neurons, respectively, and an output layer with two outputs. All the layers except the output layer used the ReLU activation function and regularization via a dropout probability of 40%. A cross-entropy loss function and the Adam optimizer (with a learning rate of 0.0003) were used for training. The binary classification network was trained for 150 epochs with a batch size of 20 for less than five minutes (see Figure S4(a)). While the classifier was extremely accurate in classifying dead cells in the test dataset, some healthy neutrophils were misclassified as dead cells. Thus, we added an additional checkpoint that updated the classification of all dead cells based on thresholds for mean intensity, Euler number, and area of the cells and their nuclei.

We then trained a five-part classifier that used the same feature vector and consisted of an input layer with 512 inputs, four hidden layers with 256, 128, 64, and 32 neurons, respectively, and an output layer with five outputs. Once again, the ReLU activation function and regularization via a dropout probability of 40% were used for all layers except the output. A cross-entropy loss function and the Adam optimizer (with a learning rate of 0.0003) were used for training. The binary classification network was trained 150 epochs with a batch size of 20 for less than five minutes (see Figure S4(b)). The classifier was tested on the test dataset, and specificity and sensitivity for each class were computed.

### 4.7. Automatic Cell Counting

In order to obtain WBC counts from each sample, the RGB image tiles (consisting of the grayscale image and the postprocessed segmentation masks) were stitched to generate a single wide-field image using the Grid/Collection stitching plugin (that was also used to stitch the virtually stained image tiles). Counting WBCs in the wide-field image rather than the image tiles avoids multiple counting of WBCs in overlapping tiles and minimizes misclassifications arising from incomplete cells on tile edges. WBCs from the wide-field image were cropped (into 224×224 pixel patches) and classified into “healthy” or “dead” cells. All the dead cells were passed through the checkpoint, and all the healthy cells obtained after this step were then passed to the five-part classifier. The number of cells of each type was counted, and the output was overlaid on the stitched virtually stained image to provide additional information for visual hematology analysis.

### 4.8. Fixing and Staining of Blood Smear Samples

After UV imaging, blood smear samples were first fixed using methanol (Thermo Fisher Scientific) for 7 minutes and stained in May-Grünwald solution (MG500; Sigma-Aldrich) for 15 minutes. After a brief rinse, the samples were put in a 1 : 10 diluted Giemsa stain solution (GS500; Sigma-Aldrich) for 20 minutes. Samples were then washed in a phosphate buffer solution (pH 6.6) and air-dried for bright-field microscopy as explained in Ref. [21].

## Data Availability

Files to support this study can be accessed through the associated Open Science Framework (doi:10.17605/OSF.IO/AYW4J).

## Supplementary Materials

Supplementary Materials Figure S1: Enhanced virtual staining of eosinophils after segmentation and classification. Figure S2: Training and validation losses for the generator for virtual staining. Figure S3: Training and validation losses for cellular and nuclear segmentation. Figure S4: Training losses for fully connected networks for classification. Table S1: Comparison of segmentation models with U-Net.
