# Supplementary material for: Virtual Staining, Segmentation, and Classification of Blood Smears for Label-Free Hematology Analysis
Source: BME Front. 2022 Jul 1;2022:9853606. doi: 10.34133/2022/9853606 (PMC10521747; doi:10.34133/2022/9853606)
Supplement: Supplementary Materials — Figure S1: Enhanced virtual staining of eosinophils after segmentation and classification. Figure S2: Training and validation losses for the generator for virtual staining. Figure S3: Training and validation losses for cellular and nuclear segmentation. Figure S4: Training losses for fully connected networks for classification. Table S1: Comparison of segmentation models with U-Net. [file 9853606.f1.docx]

Supplementary Information

Virtual Staining, Segmentation and Classification of Blood Smears for Label-free Hematology Analysis

Nischita Kaza^1^, Ashkan Ojaghi ^2^, Francisco E. Robles ^2*^

^1^School of Electrical and Computer Engineering, Georgia Institute of Technology, Atlanta, Georgia, USA

^2^Wallace H. Coulter Department of Biomedical Engineering, Georgia Institute of Technology and Emory University, Atlanta, Georgia, USA

^*^Corresponding author. Email: robles@gatech.edu


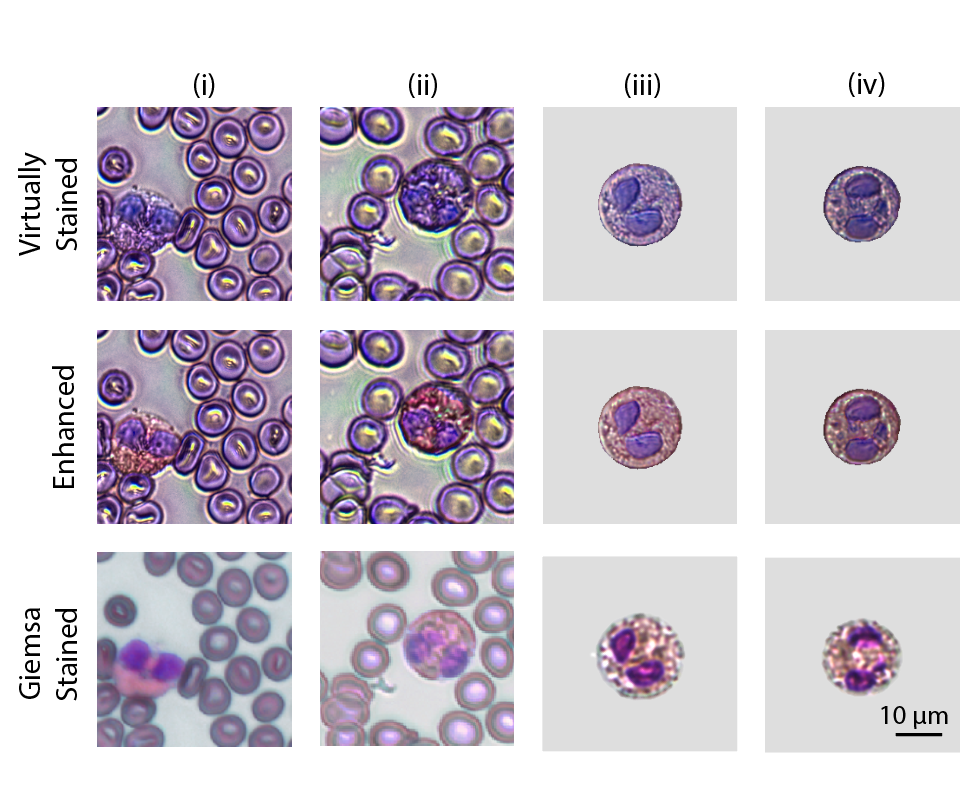


**Figure S1. Enhanced virtual staining of eosinophils (in smears (i and ii) and isolated (iii and iv)) after segmentation and classification.** The virtually stained images (first row) were converted to the HSV colors space; a value of 0.2 was added to the hue channel of the cytoplasm (the cytoplasm mask was obtained by subtracting the nuclear mask from the cellular mask) and a value of 0.05 was added to the hue channel of the nucleus of the cell classified as an eosinophil. The images were the converted back to the RGB color space (second row).


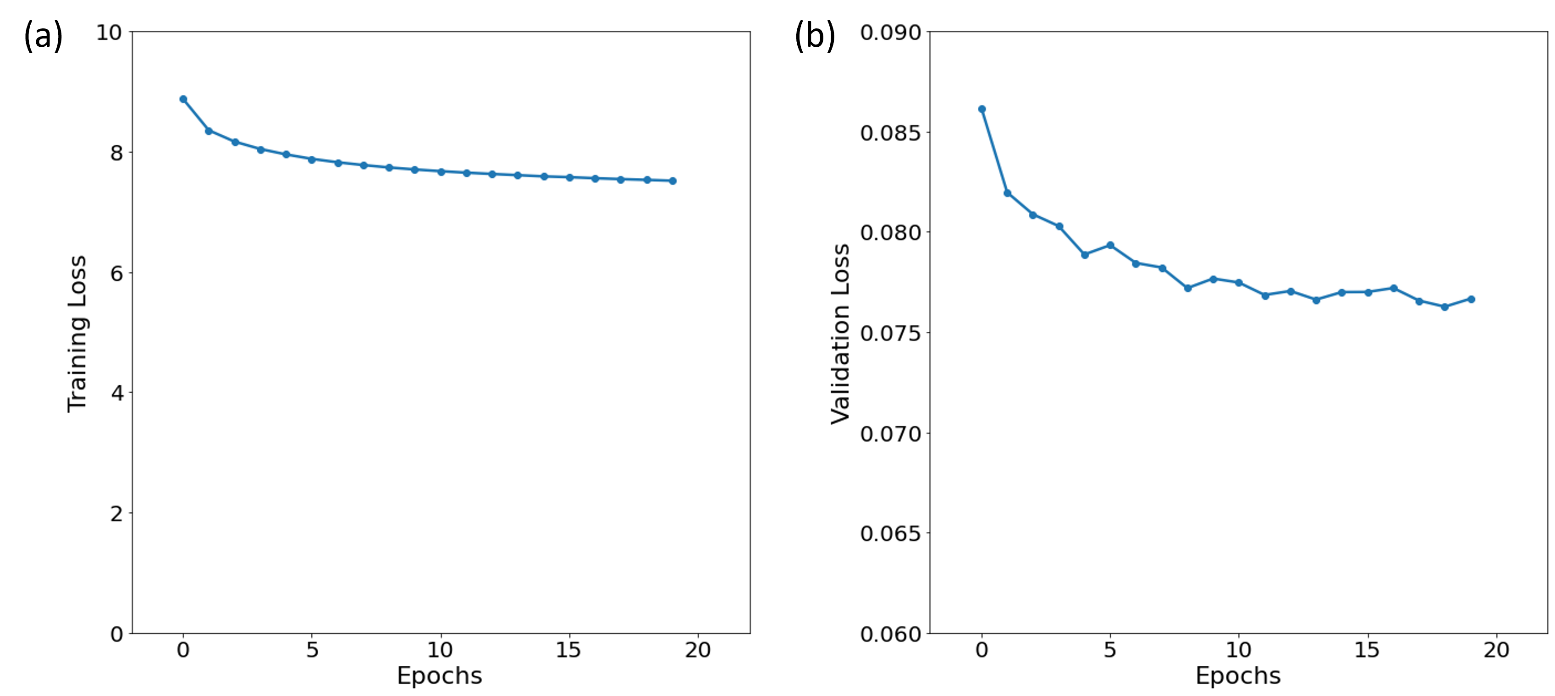


**Figure S2. Training and validation losses for the generator for virtual staining:** (a) Generator training loss over 20 epochs (b) *L*1 loss computed on the validation set over 20 epochs.


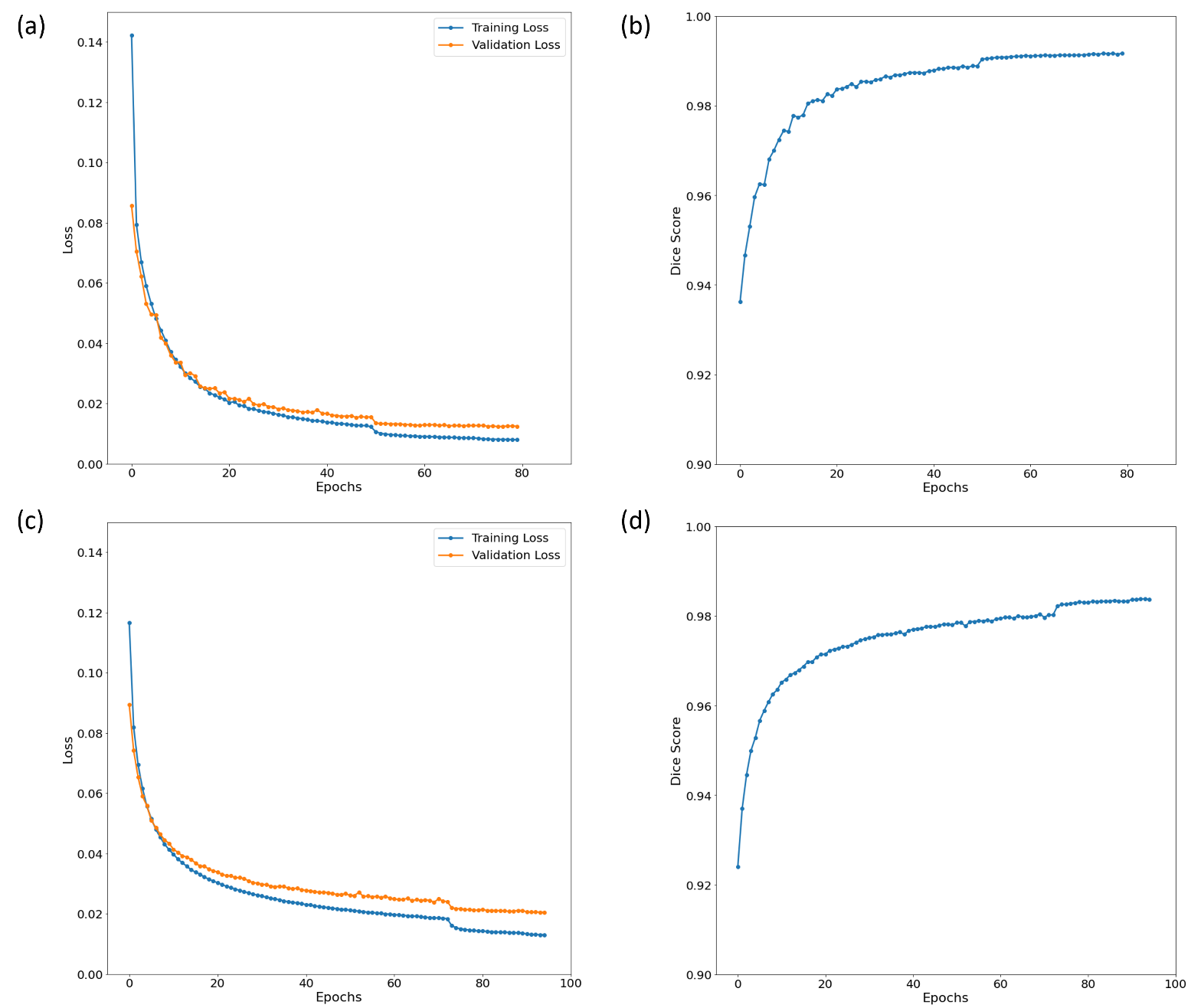


**Figure S3. Training and validation losses for cellular and nuclear segmentation:** (a) Training and validation loss for cell segmentation (b) Dice score computed on the validation set for cell segmentation (c) Training and validation loss for nuclear segmentation (d) Dice score computed on the validation set for nuclear segmentation


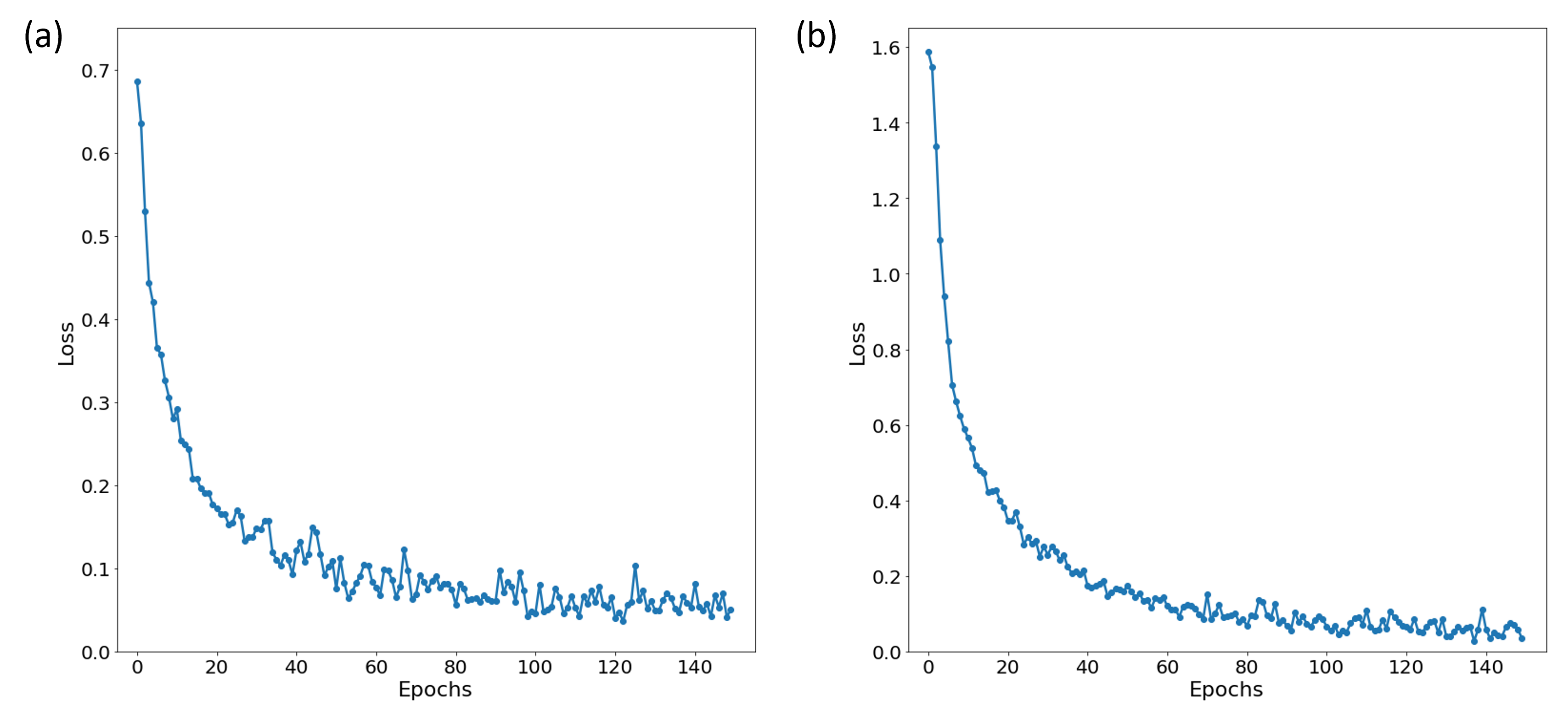


**Figure S4. Training losses for fully connected networks for classification:** (a) Training loss for binary classifier classifying cells as `healthy' or `dead' (b) Training loss for five-part WBC classifier

*Comparison of Segmentation models with existing models:*

| Model | Cellular Segmentation | Nuclear Segmentation |
| --- | --- | --- |
| Proposed model (see Fig. 6(b)) | 0.9899 | 0.9718 |
| U-Net (trained with Cross entropy loss) | 0.9926 | 0.9828 |
| U-Net (trained with cross entropy & dice loss) | 0.9929 | 0.9816 |

**Table S1: Comparison of segmentation models with U-Net:** Dice score averaged across the same test dataset for each model

The performance of our segmentation model was compared to the U-Net [48]. The only modification to the architecture was the use of padded convolutions to have inputs and outputs of the same size (for a fair comparison with our models) and the introduction of batch normalization to stabilize training. The U-Net was trained for cellular and nuclear segmentation using the cross-entropy loss as per Ref. [48], and this was compared with models trained using the combination of the cross-entropy and the dice-loss (same as the loss function used to train the proposed segmentation models to provide a fairer comparison of the architecture). Each of the models was trained for 50 epochs, and the best model (chosen on the basis of the lowest validation loss) was used for testing. While the U-Net does results in slightly better segmentation of both cells and nuclei, this comes as the cost of additional parameters and slightly longer inference times. Further, this small difference in segmentation performance has little impact on further processing.
